# Novel approach to enhancing brain DHA uptake: the role of nannochloropsis microalgae extract

**DOI:** 10.3389/fnut.2025.1573310

**Published:** 2025-07-09

**Authors:** Kunpeng Ma, Shuhui Xie, Ying Zhang, Haixiang Liu, Wei Yu, Peihua Chen

**Affiliations:** ^1^Interdisciplinary Center for Brain Information, the Brain Cognition and Brain Disease Institute, Shenzhen Institutes of Advanced Technology, Chinese Academy of Sciences, Shenzhen, China; ^2^Sino-Danish College, University of Chinese Academy of Sciences, Beijing, China; ^3^Xiaozao Technology (Anji) Co., Ltd, Anji, China; ^4^CAS Key Laboratory of Brain Connectome and Manipulation, Shenzhen-Hong Kong Institute of Brain Science, Shenzhen Institutes of Advanced Technology, Chinese Academy of Sciences, Shenzhen, China

**Keywords:** omega-3 fatty acids, nannochloropsis microalgae, blood-brain barrier, LDGTS, DHA, Mfsd2a

## Abstract

**Background:**

Docosahexaenoic acid (DHA) plays a pivotal role in sustaining the normal function of the human brain and preventing metabolic and neurodegenerative disorders. Despite its significance, the bioavailability of DHA from current supplements is suboptimal due to the limited absorption capacity of the adult brain. Recent studies have highlighted the role of Mfsd2a transporter in facilitating brain DHA uptake when DHA is esterified to lyso-phosphatidylcholine (LPC).

**Objective:**

Lyso-diacylglyceryltrimethylhomoserine (LDGTS), a betaine lipid that resembles LPC in structure and is abundant in Nannochloropsis microalgae oil, presents a promising method to enhance DHA absorption. This study aimed to examine whether LDGTS-containing microalgae oil can promote brain DHA uptake.

**Methods:**

Seven groups of mice were fed different lipid formulations alongside their regular diet. After 15 days, tissue and organ samples were collected for lipid extraction and fatty acid analysis. Brain DHA uptake was quantified and compared across groups.

**Results:**

The mice administered a combination of microalgae oil and triacylglycerol (TAG)-DHA showed a significant increase in brain DHA uptake compared to controls. This effect was not observed with TAG-DHA alone, microalgae oil alone, or krill oil. The facilitation of DHA incorporation was accompanied by a notable enhancement of pathways related to cell growth and retinol metabolism in the brain, while pathways associated with cellular apoptosis and inflammation were downregulated.

**Conclusion:**

These findings suggest that the inclusion of LDGTS-rich microalgae oil in DHA supplementation may offer a novel and effective strategy for improving the bioavailability of DHA in the brain.

## 1 Introduction

The human body's capacity to synthesize docosahexaenoic acid (DHA) is minimal, which underscores the importance of dietary intake as the primary means of obtaining this essential fatty acid (1). The concentration of DHA in plasma is directly related to an individual's consumption of DHA-rich supplements or foods, and the levels of DHA in peripheral tissues (e.g., the heart, kidneys, and muscles) are associated with plasma phospholipid DHA levels (2). Despite the clear benefits of DHA uptake in the brain, the brain's DHA levels remain relatively stable and do not significantly fluctuate with changes in blood DHA levels (2, 3). Clinical trials have failed to demonstrate a curative effect from the use of conventional DHA supplements (e.g., fish oil, krill oil, or purified DHA) in patients with neurological conditions like Alzheimer‘s, Huntington's, and schizophrenia (4–6). This lack of therapeutic success is likely due to the inability of these supplements to sufficiently enrich plasma levels of LPC-DHA, the preferred form for brain uptake (7).

LPC has been identified as a primary source of brain DHA (8–10). The transport of LPC across the luminal plasma membrane of the blood-brain barrier (BBB) is facilitated by a specific transporter, the major facilitator superfamily domain-containing protein 2a (Mfsd2a) (8). Although dietary administration of LPC-DHA has been shown to enhance brain DHA uptake in murine models, this compound is rarely present in natural food sources or commercial dietary supplements (9). Current methods for producing LPC-DHA involve an enzymatic process using krill oil (rich in PC-DHA) followed by multiple biochemical reactions (11, 12). However, this approach relies on lipase derived from *Mucor miehei*, which not only substantially increases production costs but also lacks comprehensive safety evaluation for food applications.

Mfsd2a has been found to transport both LPC-fatty acids and structurally analogous LPC-like molecules. This transport mechanism depends on two key structural features: a zwitterionic headgroup and a minimum 14-carbon hydrophobic tail (13, 14). Guided by this principle, we screened potential DHA carriers from the polar lipid-rich extract of Nannochloropsis microalgae metabolites. Nannochloropsis, a genus within the Eustigmatophyceae family of microalgae, is recognized for its ability to produce valuable lipids and has garnered significant interest due to its rapid growth, high lipid content, and natural synthesis of EPA (15, 16). Despite the low concentrations of LPC, a betaine lipid known as lyso-diacylglyceryltrimethylhomoserine (LDGTS) is present in substantial amounts in Nannochloropsis extracts (17, 18). LDGTS is distinguished by its zwitterionic headgroup at the sn-3 position and a single hydrophobic hydrocarbon tail located at either the sn-1 or sn-2 position, with the other position being unacylated. With a carbon chain exceeding 14 carbons, LDGTS is predicted to cross the BBB with the Mfsd2a transporter, a mechanism employed by LPC. Furthermore, DGTS/LDGTS in microalgae exhibits similarities to PC/LPC in plants in the synthesis and remodeling pathways (18).

These unique characteristics of LDGTS suggest its potential to facilitate DHA transport across the BBB via Mfsd2a—a novel hypothesis we proposed that remains experimentally unverified. The primary objective of this study was to investigate whether co-administration of DHA with LDGTS-rich Nannochloropsis extract could enhance brain DHA uptake in mice, thereby validating Nannochloropsis microalgae oil as a natural alternative to LPC.

## 2 Materials and methods

### 2.1 Algaculture and algae oil extraction

The cultivation of Nannochloropsis gaditana strains adapted to Fangchenggang's local conditions through independent breeding involved stepwise amplification, beginning with 5L triangular flasks, progressing to tubular photobioreactors, and finally to outdoor open raceway ponds. During the cultivation period, nitrogen and phosphorus elements were added, and the cultivation cycle lasted about 10 days before harvesting. A disc stack centrifuge was used to separate the algal liquid to obtain concentrated algal liquid, which was then spray-dried to obtain algal powder. The powder was then processed through ethanol extraction, filtration, and concentration to produce algae oil (Table 1).

**Table 1:** Contents in nannochloropsis gaditana oil.

**Head group**	**Content (mg/g)^a^**	**FA with DGTS**	**Content (mg/g)^a^**
DGTS	7.86	DGTS 31:1|DGTS 14:0_17:1	0.05
LDGTS	29.72	DGTS 32:1|DGTS 16:0_16:1	0.82
FFA	62.39	DGTS 32:2|DGTS 16:1_16:1	0.28
DG	43.18	DGTS 34:1|DGTS 16:0_18:1	0.08
MG	74.06	DGTS 34:2|DGTS 16:1_18:1	0.17
TG	19.04	DGTS 34:3|DGTS 16:1_18:2	0.10
Cer	3.24	DGTS 36:5|DGTS 16:0_20:5	0.58
MGDG	15.33	DGTS 36:6|DGTS 16:1_20:5	0.41
DGDG	12.05	DGTS 38:7|DGTS 18:2_20:5	0.11
SQDG	20.54	DGTS 40:10|DGTS 20:5_20:5	4.35
DGGA	0.38	DGTS 40:9|DGTS 20:4_20:5	0.91
CE	2.73	**FA with LDGTS**	**Content (mg/g)^a^**
CL	1.65	LDGTS 14:0	0.61
LPA	0.90	LDGTS 15:0	0.14
LPE	0.09	LDGTS 16:0	3.50
LPG	1.06	LDGTS 16:1	3.53
LPC	1.72	LDGTS 16:2	0.19
LPI	0.14	LDGTS 17:0	0.09
PE	3.89	LDGTS 17:2	0.09
PG	13.98	LDGTS 18:0	0.04
PI	4.72	LDGTS 18:1	0.93
PC	29.18	LDGTS 18:2	0.46
PI-Cer	2.27	LDGTS 19:5	0.13
**Sum**	350.12	LDGTS 20:5	20.01

FFA, free fatty acid; FA, fatty acid.

a The content is represented as the total weight of the head group connected with fatty acid chains, except for that of FFA which represented the total amount of non-esterified fatty acid.

### 2.2 Diets and treatment

Animal care and experimental protocols were approved by the Animal Care and Use Committees at the Shenzhen Institute of Advanced Technology, Chinese Academy of Sciences. A total of 40 12-week-old C57BL6/J male mice (Hunan SJA) were randomly divided into eight groups, with five mice per group, and housed in a specific pathogen-free (SPF) facility. After a one-month acclimatization period, the mice were administered the test substances via gavage. In addition to their regular diet, each group of mice was also given 80 μl of either pure corn oil or a corn oil solution containing various substances daily (Table 2). The mice in the blank control group (BC) were fed with pure corn oil. The mice in test groups T1 and T2 received experimental diets containing microalgae oil-TAG-DHA mixtures at precisely formulated ratios: T1 received 0.72 mg microalgae oil with 1.43 mg TAG-DHA (1:2 ratio), while T2 received 1.43 mg microalgae oil with 1.43 mg TAG-DHA (1:1 ratio). The TAG-DHA (Cat.#ADT700BW, Guangxi Xiaozao Agricultural Technology Co., Ltd, China) provided a standardized ~1 mg DHA dose per administration. This dose-ranging design enabled systematic comparison of DHA absorption efficiency between the two formulation ratios. The mice in the comparison 1 group (C1) or comparison 2 group (C2) were given 0.72 mg or 1.43 mg of microalgae oil alone. The mice in comparison 3 group were fed with TAG-DHA (1.43 mg). To compare the effects of the microalgae oil and DHA mixture with other available DHA supplements, we fed the mice in group 4 (C4) with krill oil (11.2 mg, containing ~1 mg of DHA; Cat.#KO2306009, Shandong Kangjing Marine Bioengineering Co., Ltd, China). One mouse in test 2 (T2) group died on the eighth day due to an error in gavage procedure. After 15 days of feeding, the mice were anesthetized with isoflurane, and tissue and organ samples were collected and frozen in a dry ice-ethanol mixture. The samples were then transferred to a −80°C freezer for further analysis.

**Table 2:** Composition of daily gavage.

**Group**	**Corn oil**	**Material for mouse feeding (/per mouse)**
Blank control (BC)	80 μl	–
Test 1 (T1)	80 μl	Microalgae oil 0.72 mg + TAG-DHA 1.43 mg^a^
Test 2 (T2)	80 μl	Microalgae oil 1.43 mg + TAG-DHA 1.43 mg^a^
Comparison 1 (C1)	80 μl	Microalgae oil 0.72 mg
Comparison 2 (C2)	80 μl	Microalgae oil 1.43 mg
Comparison 3 (C3)	80 μl	TAG-DHA 1.43 mg^a^
Comparison 4 (C4)	80 μl	Krill oil 11.2 mg^b^

a 1.43 mg of TAG-DHA contains ~1 mg of DHA.

b 11.2 mg krill oil contains ~1 mg of DHA and ~6.3 mg of phospholipid.

### 2.3 Lipid extraction and fatty acid analysis

For each organ preserved as mentioned above, 20–50 mg of sample was cut and placed into a new polyethylene tube. Lipid extraction was conducted following a standard protocol described by Ivanova et al. (19). To the sample was added 800 μl of cold 0.1 N HCl:CH_3_OH (1:1, Cat.# C1120150023 and Cat.#C0690110225, Nanjing Reagent, China). The samples were then homogenized for 1 min using a multi-channel grinder. Afterward, 400 μl of ice-cold CHCl_3_ (Cat.#C0761520225, Nanjing Reagent) was added, and the tube was vortexed for about 1 min and centrifuged for 5 min at 4°C at 18,000 g. Following centrifugation, the supernatant was discarded, and the remaining lipid was placed in an environment at 105°C and baked for about 40 min until completely dry. Once cooled to room temperature, the dried sample was weighted and placed in a hydrolysis tube, followed by the addition of 0.25 ml of internal standard solution (C11:0 methyl ester in methanol, 2 mg/ml; Cat.#47147; Millipore). A 5 ml NaOH-methanol solution (2% W/V; Cat.#C0131510226, Nanjing Reagent) was added to the hydrolysis tube, which was then flushed with nitrogen for 30 sec and sealed before being placed in a water bath at 80°C for hydrolysis for about 40 min. After cooling, a 10 mL sulfuric acid-methanol solution (5% W/V; Cat.# C0680150225, Nanjing Reagent) was added to the tube, which was flushed with nitrogen for another 30 sec, sealed, and placed in a boiling water bath for a reaction time of 20 min. After cooling, 5 ml of n-hexane (Cat.# C0760512224, HPLC, Nanjing Reagent) was added to the hydrolysis tube, followed by vortex mixing for 1 min. Then, 15 ml of water was added, and the mixture was allowed to stand for about 5 min. The upper n-hexane layer (1 ml) was taken and filtered into a sample vial for gas chromatography (GC) injection, and the peak areas of each fatty acid were recorded. A mixed standard solution of fatty acid methyl esters of known concentrations (Cat.#SMB00937, Millipore) was also injected into the GC for analysis, and the peak areas of each fatty acid were recorded. The fatty acid content in the sample was calculated by comparing the peak areas to a standard curve of varying concentrations, and the calculated results were normalized to the blank control (BC) group.

### 2.4 Lipidomic analysis

The lipidomic data analysis was performed by Shanghai Luming Biological Technology Co., Ltd (Shanghai, China). An ACQUITY UPLC I-Class plus (Waters Corporation, Milford, USA) fitted with a Q-Exactive mass spectrometer and equipped with a heated electrospray ionization (ESI) source (ThermoFisher, USA) was used to analyze the metabolic profiling in both ESI positive and ESI negative ion modes.

### 2.5 Protein extraction and digestion

Frozen tissue samples (~100 mg) were rapidly pulverized into a fine, uniform powder in liquid nitrogen and then thoroughly homogenized in 1 ml of phenol extraction buffer (Cat.#T0250, Solarbio, China). Subsequently, 1 ml of saturated phenol solution with Tris-HCl (pH 7.5; Cat.#B548124, Sangon Biotech, China) was added. The mixture was gently agitated multiple times to ensure thorough blending and then refrigerated at 4°C for 30 min to allow for the partitioning of components. The upper phenolic liquid, rich in extracted proteins, was carefully separated from the aqueous phase by centrifugation at 7,100 g for 10 min at 4°C. The supernatant was then transferred to a new tube and mixed with five volumes of pre-chilled 0.1 M ammonium acetate-methanol solution (Cat.# C0410580223, Nanjing Reagent). After overnight incubation at −20°C, the mixture was centrifuged at 12,000 g for 10 min at 4°C to pellet the precipitated proteins. The wash process involved resuspending the pellet in pre-chilled methanol twice, followed by two rounds of resuspension in ice-cold acetone (Cat.# C0720110223, Nanjing Reagent). Each resuspension was followed by centrifugation to purify the protein pellet further. Once the final pellet was collected, it was air-dried to remove any residual solvents and then resuspended in 300 μl of lysate solution. The sample was then incubated at room temperature for 3 h to ensure complete solubilization of the proteins. Following this incubation, the solution was centrifuged again to remove any remaining insoluble material, leaving behind a supernatant that contained the total extractable protein fraction. The concentration of total protein in the supernatant was determined using the bicinchoninic acid (BCA) assay (Cat.#23225, ThermoFisher). Based on the quantified protein concentrations, equal amounts of protein were aliquoted from each sample. The samples from different groups were then diluted to achieve uniform concentrations and volumes. An appropriate volume of 25 mM dithiothreitol (DTT; Cat.# 1064272, Adamas-beta) was added to achieve a final concentration of ~5 mM. The samples were then incubated at 55°C for a duration of 30–60 min to facilitate the reduction of disulfide bonds. Subsequently, iodoacetamide (Cat.#I6125, Millipore) was introduced to the mixture with a final concentration of about 10 mM. The samples were protected from light and allowed to react at room temperature for 15–30 min to alkylate the reduced cysteine residues. Following the alkylation step, precooled acetone, in a volume 6 times that of the protein solution, was added to the mixture to precipitate the proteins. The samples were then placed at −20°C overnight. After precipitation, the samples were then centrifuged at 8,000 g for 10 min at 4°C to collect the protein precipitate. Based on the protein content, an appropriate volume of TPCK-treated trypsin (Cat.#A003740, Sangon Biotech) was added, following a protein to enzyme ratio of 50:1 (mass/mass) to redissolve the protein precipitate. The samples were then incubated at 37°C for 12 h to allow for enzymatic digestion of the proteins. Finally, the digested samples were lyophilized to remove the solvent, yielding the processed protein samples ready for subsequent analysis or applications.

### 2.6 Proteomic analysis

Tandem Mass Tag (TMT) labeling process and proteomic data analysis were expertly conducted by Shanghai Luming Biological Technology Co., Ltd (Shanghai, China), and Proteome Discoverer (v.2.4) was used to conduct a comprehensive search against the Uniprot Mus Musculus protein database for all raw mass spectrometry data. The database search was configured with the specificity of trypsin digestion. Fixed modifications were accounted for by including alkylation of cysteine residues in the search parameters. For the protein quantification aspect of the analysis, the TMT method was utilized, which allows for the relative quantification of proteins across multiple samples. A global false discovery rate (FDR) was set to 0.01 and protein groups considered for quantification required at least 1 peptide. A total of 6,306 proteins expressed were identified as belonging to the proteome of mouse brains in this study. The thresholds of fold change (>1.2 or <0.83) and *p*-value <0.05 were used to identify differentially expressed proteins (DEPs).

### 2.7 Statistical analysis

**Figure 1:**
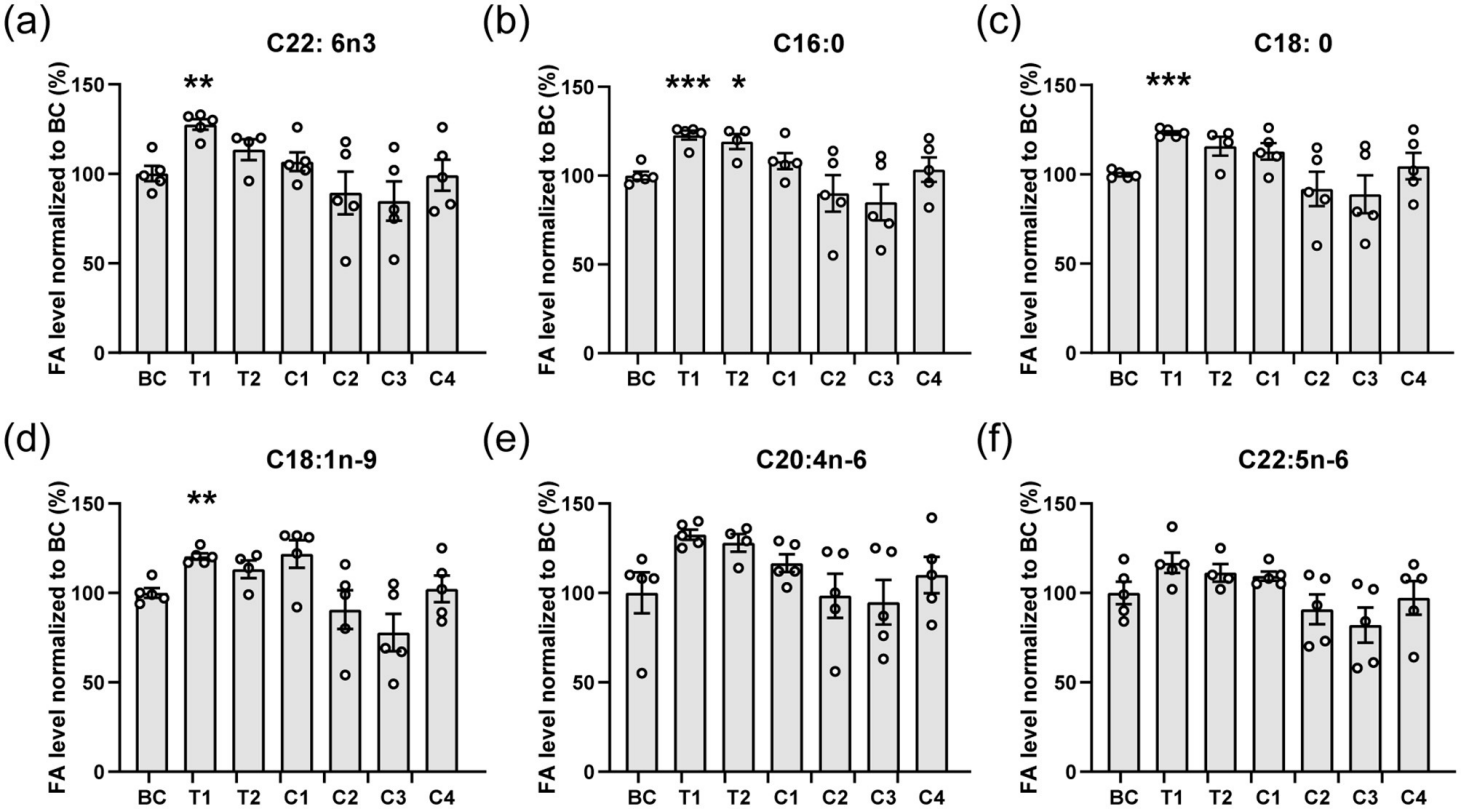
Main fatty acids composition of the brain. **(a–f)** The main fatty acids including C22: 6n3 (DHA, **a**), C16:0 **(b)**, C18:0 **(c)**, C18:1n-9 **(d)**, C20:4n-6 **(e)**, and C22:5n-6 **(f)** from brains of mice were analyzed. The contents of fatty acids are normalized to the blank control (BC). All data are mean ± SEM. ***p < 0.001, **p < 0.01, and *p < 0.05 compared to BC. Statistical significance was determined using Brown-Forsythe and Welch ANOVA tests with Dunnett's T3 correct for all panels.

**Figure 2:**
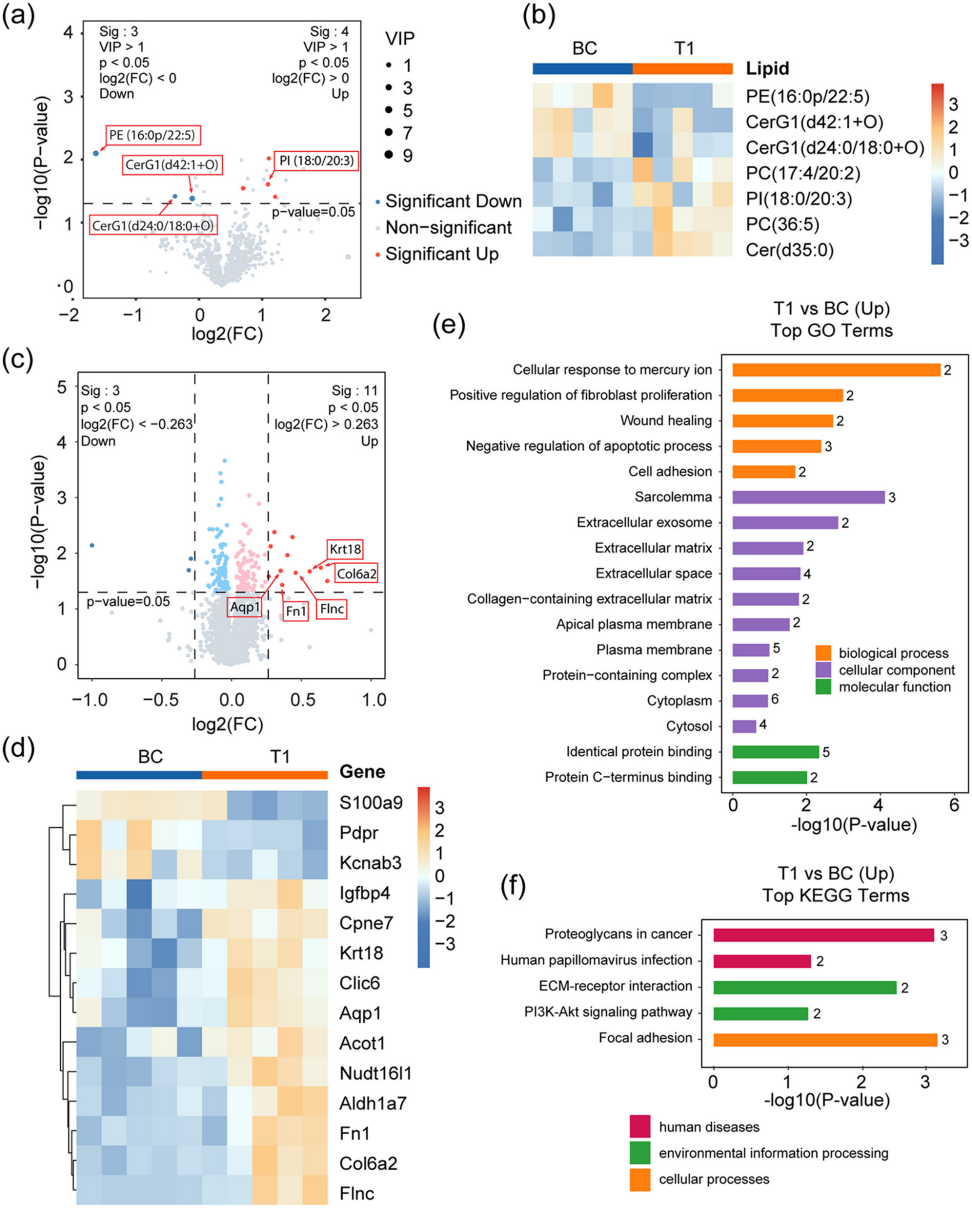
Lipidomic and proteomic analyses of T1 against BC. **(a)** Volcano plot showing the significantly changed lipid metabolites in T1 compared to BC. Red and blue dots represent up- and down-regulated metabolites respectively, with a threshold of *p*-value <0.05, VIP >1, and FC >1; Other detected metabolites are drawn as gray dots. **(b)** Heatmap representing the three down-regulated and four up-regulated metabolites in BC and T1 groups. **(c)** Volcano plot showing the significantly changed proteins in T1 compared to BC. Dark red and dark blue dots mark significantly up- and down-regulated proteins respectively, with a threshold of *p*-value <0.05 and absolute value of log2(FC) > 0.263. Light red and light blue dots represent up- and down-regulated proteins respectively, with a threshold of p-value <0.05 but without satisfying the FC threshold above. Other proteins are dotted gray. **(d)** Heatmap representing the three down-regulated (dark red dots in d) and 11 up-regulated proteins (dark blue dots in d) in T1 against BC. **(e, f)** Up-regulated pathways identified by GO **(e)** and KEGG **(f)** enrichment analysis. *p*-values represent Benjamini-Hochberg FDR-corrected values.

The data are presented as means ± SEM, with statistical significance thresholds set at ^*^p < 0.05, ^**^p < 0.01, and ^***^p < 0.001 for all analyses. Group comparisons in Figures 1a–f were performed using Welch's ANOVA to account for potential variance heterogeneity, followed by Dunnett's T3 post-hoc tests for multiple comparisons against the baseline control (BC). For Figures 2a, d, unpaired two-tailed Student's *t*-tests were employed to assess differences in metabolite levels and protein expression, while Figures 2c, f utilized hypergeometric tests with false discovery rate (FDR) adjustment for enrichment analyses.

## 3 Results

### 3.1 LDGTS is enriched in nannochloropsis microalgae oil

**Figure 3:**
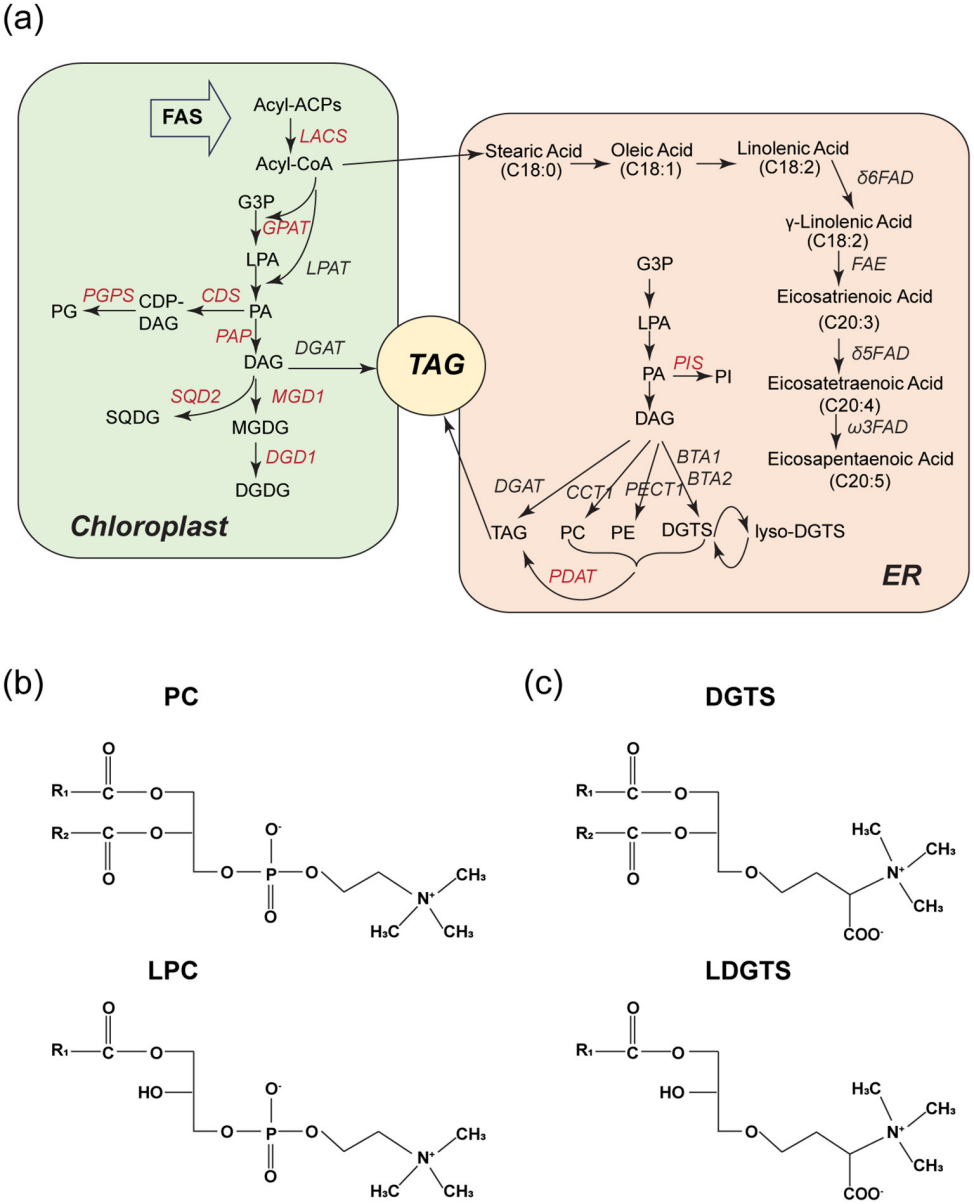
LDGTS is enriched in Nannochloropsis microalgae metabolites and has structural similarity to LPC. **(a)** Schematic of reactions involved in lipid metabolism in Nannochloropsis microalgae during TAG accumulation. Enzymes are in italics; for those shown in red further evidence is needed. DAG is the primary source for the synthesis of DGTS and LDGTS. The DAG pool is predominantly accumulated through the Kennedy pathway, which initiates with the transacylation of glycerol-3-phosphate (G3P) at the sn-1 position to form lyso-phosphatidic acid (LPA) followed by an additional transacylation at the sn-2 position to form phosphatidic acid (PA). Subsequently, PA is dephosphorylated at the sn-3 position, culminating in the production of DAG. The biosynthesis pathway of eicosapentaenoic acid (EPA, C20:5) starts from stearic acid (C18:0) and encompasses a sequence of reactions catalyzed by fatty acid desaturases and a fatty acid elongase. **(b, c)** Structures of PC, LPC (b), and DGTS, LDGTS **(c)**. They all have a zwitterionic headgroup. Both LPC and LDGTS have only one hydrophobic hydrocarbon tail. If the carbon chain of LDGTS exceeds 14 carbons in length, it is anticipated to cross the blood-brain barrier via the Mfsd2a transporter, a mechanism utilized by LPC.

Nannochloropsis gaditana produces a diverse range of polar lipids through its metabolic pathways (Figure 3a). Among these, LDGTS is of particular interest to us due to its high content and potential ability to cross the BBB, as suggested by its structural similarity to LPC and the refined rule proposed by previous research (Figures 3b, c) (13, 14). LDGTS contained fatty acids ranging from saturated C14 to unsaturated C20:5 (EPA) esterified to the glycerol backbone, with EPA being the most prevalent, accounting for up to 67% of total LDGTS (the concentration of LDGTS-EPA reaches ~20 mg/g, with other FAs totaling ~10 mg/g, Table 1). LDGTS is mainly synthesized from the pool of diacylglycerol (DAG), and EPA biosynthesis starts from stearic acid (C18:0) and encompasses a sequence of reactions catalyzed by fatty acid desaturases and a fatty acid elongase (Figure 3a). Notably, Nannochloropsis species lack Δ4-desaturase and the elongase ELOVL2 (Elongation of Very Long-Chain Fatty Acids Protein 2), which are essential for the final conversion of EPA to DHA (20, 21). Consequently, Nannochloropsis-derived microalgae extracts are completely devoid of endogenous DHA.

Given that the metabolic activity of Nannochloropsis microalgae is influenced by environmental conditions, the contents of LDGTS and other components in the extract vary monthly. To represent an average level of annual production, four batches of extract, independently collected in January, March, June, and December, were mixed in a 1:1:1:1 volume ratio to create the final microalgae oil which was used for the following experiment. Liquid chromatography coupled to tandem mass spectrometry (LC-MS/MS) analysis revealed that LDGTS was present at a content of 29.72 mg/g (W/W) in the microalgae oil, significantly higher than that of other lyso-phospholipids, including lyso-phosphatidic acid (LPA), lyso-phosphatidylethanolamine (LPE), lyso-phosphatidylglycerol (LPG), LPC, and lyso-phosphatidylinositol (LPI). The content of the microalgae oil is provided in Table 1.

### 3.2 Dietary intake of the mixture of LDGTS-containing microalgae oil and TAG-DHA to enhance DHA incorporation by the brains of mice

To assess the ability of LDGTS-rich microalgae oil to deliver DHA to the brain, we established seven groups of mice administered different lipid formulations in addition to their regular diet (Table 2). The mice in the blank control group (BC) were fed with pure corn oil. The mice in the test 1 group (T1) or test 2 group (T2) were fed mixtures containing 0.72 mg or 1.43 mg of microalgae oil with TAG-DHA (1.43 mg, containing ~1 mg of DHA), respectively. The mice in the comparison 1 group (C1) or comparison 2 group (C2) were given 0.72 mg or 1.43 mg of microalgae oil alone, respectively. The mice in the comparison 3 group (C3) were fed with TAG-DHA (1.43 mg). To compare the effects of the microalgae oil and DHA mixture with other available DHA supplements, we fed the mice in group 4 (C4) with krill oil (11.2 mg, containing ~1 mg of DHA).

Following the 15-day feeding period, we analyzed the DHA and other fatty acid content in mouse brains. Comparison between group C3 (1.43 mg TAG-DHA) and BC revealed that TAG-DHA intake alone did not enhance brain DHA uptake (C22:6n−3, Figure 1a), consistent with prior reports (9). As anticipated, mice fed exclusively with microalgae oil (groups C1 and C2) also showed no increase in DHA accumulation, as the microalgae oil itself lacks endogenous DHA. Interestingly, a simple physical mixture of Nannochloropsis microalgae oil and TAG-DHA at a specific ratio (T1) significantly promoted DHA accumulation in the mouse brains. Additionally, other major fatty acids, including palmitic acid (C16:0, Figure 1b), stearic acid (C18:0, Figure 1c), and oleic acid (C18:1n−9, Figure 1d), were also significantly increased in group T1. We also evaluated commercially available krill oil (C4), which contains DHA in the form of PC-DHA (22). Since PC-DHA is less efficient at crossing the blood-brain barrier (BBB), no increase in brain DHA concentration was detected in this group. These results confirmed that Nannochloropsis microalgae oil can indeed facilitate DHA uptake by the brain.

### 3.3 Dietary supply of microalgae oil and TAG-DHA regulates lipid metabolism and protein expression within the brain

The T1 group displayed elevated levels of fatty acids in brain tissue following the administration of a microalgae oil and TAG-DHA mixture. To comprehensively investigate the resulting alterations in lipid metabolism and protein expression, we performed comparative lipidomic and proteomic analyses between the T1 and BC groups using brain tissue samples.

Differential lipid analysis identified significant variations between the two groups. Figures 2a, b present these differentially expressed lipids as volcano plots and heatmaps, respectively. Notably, three lipid species exhibited reduced levels, with PE (16:0p/22:5) showing the most pronounced decrease. This phospholipid contains docosapentaenoic acid (DPA, C22:5), a direct precursor of DHA (C22:6), which undergoes further desaturation and elongation via enzymes such as Δ4-desaturase to form DHA (23). Under physiological conditions, DHA deficiency can lead to DPA accumulation, potentially compromising membrane fluidity (24, 25). And plasma DPA and DHA levels tend to be negatively associated (26–28). Additionally, two hexosylceramides (CerG1)—CerG1(d24:0/18:0+O) and CerG1(d42:1+O)—known to act as neuroinflammatory signaling lipids by activating glial pro-inflammatory pathways (29), were significantly reduced. This decrease may result from DHA-mediated inhibition of ceramide synthase (30, 31), further supporting DHA's anti-inflammatory role in the brain. Conversely, four lipid species were elevated in the T1 group. One of these, PI (18:0/20:3), contains eicosatrienoic acid (C20:3), a direct metabolic precursor of the pro-inflammatory mediator arachidonic acid (C20:4). The accumulation of eicosatrienoic acid may reflect DHA-mediated suppression of arachidonic acid synthesis. Given that arachidonic acid serves as the key substrate for multiple pro-inflammatory metabolic pathways, including the cyclooxygenase (COX) and lipoxygenase (LOX) pathways, these results further substantiate DHA's well-documented anti-inflammatory role (32). The remaining three elevated lipids showed no direct association with DHA intake.

Comparative proteomics revealed 14 differentially expressed proteins, including three downregulated and 11 upregulated candidates (Figures 2c, d). Gene Ontology (GO) enrichment analysis (Figure 2e) demonstrated that these proteins were associated with five key biological processes (Figure 2f): cellular response to mercury ion (Fn1, Aqp1), positive regulation of fibroblast proliferation (Fn1, Aqp1), wound healing (Fn1, Aqp1), inhibition of apoptosis (Krt18, Fn1, Aqp1), and promotion of cell adhesion (Fn1, Col6a2). KEGG pathway analysis further identified three proteins (Fn1, Col6a2, and Flnc) involved in cell adhesion, along with activation of the PI3K-Akt signaling pathway (Fn1, Col6a2), a critical intracellular cascade governing cell survival, growth, and proliferation (33).

Previous studies have well-established the neurological effects of DHA accumulation in the brain (34, 35). Our proteomic and lipidomic analyses corroborate these documented observations, providing robust evidence that supplementation with microalgae oil and TAG-DHA enhances DHA incorporation in the brain and induces corresponding biochemical adaptations.

## 4 Discussion

DHA, a crucial omega-3 PUFA, is integral to brain function and health, constituting a significant portion of the brain's PUFA content (36). Its role in the brain is multifaceted and includes participation in essential processes, exertion of anti-inflammatory effects, and protection against a range of neurological disorders (37, 38). A connection between reduced DHA levels in the brain and various neurological conditions, such as Alzheimer's, Parkinson's, Huntington's, schizophrenia, and depression, has been established (39–41). DHA, like other fatty acids, in the bloodstream is esterified to phospholipids, lyso-phospholipids, cholesteryl esters, and triacylglycerols (TAGs) within lipoproteins or exists in complex with albumin as a lyso-phospholipid or a non-esterified form (42). It was believed that non-esterified DHA could passively diffuse across the plasma membrane (43); however, more recent research has shed light on a more sophisticated mechanism involving lyso-phospholipids, particularly lyso-phosphatidylcholine (LPC) (8–10, 44). The transport of LPC across the luminal plasma membrane of the blood-brain barrier (BBB) is facilitated by Mfsd2a (8). This protein is notably abundant in the endothelium of the retina and brain (45, 46). Genetic ablation of Mfsd2a in mice results in a significant reduction of brain DHA levels, highlighting the critical role this transporter plays in maintaining adequate DHA levels in the brain (36, 47).

Despite the significant role of DHA uptake in the brain for the prevention of neurodegenerative and metabolic diseases, the intake of DHA by adults into the brain is extremely limited due to the BBB (2). Although many currently available DHA supplements may have certain effects on peripheral tissues and organs, these supplements struggle to demonstrate functionality within the brain under rigorous experimental scrutiny (2, 3). In 2014, Long N. Nguyen and colleagues first proposed Mfsd2a as a transporter protein at BBB to transport DHA in the form of LPC (8). They further identified the structural characteristics necessary for molecules transported by Mfsd2a in their subsequent work (13, 14). This has prompted our search for potential substances capable of this function within natural materials. We noted that the natural metabolites of Nannochloropsis microalgae are rich in LDGTS, which aligns with the structural features (17). Our results demonstrated that LDGTS-containing microalgae oil can promote the incorporation of DHA by the brain.

LDGTS is likely to increase the level of DHA in the brain through two potential pathways: First, once LDGTS-EPA (the primary form of LDGTS in microalgae oil) enters the brain, the EPA is hydrolyzed from the backbone and is subsequently converted into DHA, a transformation that has been documented in the literature (48); Second, LDGTS linked to EPA or other FAs in microalgae oil could be enzymatically converted into LDGTS-DHA before entering the brain, thereby facilitating the transport of DHA into the brain. Our results showed that microalgae oil alone, in the absence of TAG-DHA, does not significantly enhance DHA levels in the brain, indicating the minimal efficiency of EPA conversion to DHA within the brain compared to the observed increase in DHA levels. Thus, we favor the latter scenario that LDGTS-DHA forms in the circulation. However, the evidence on whether and how LDGTS in microalgae oil directly facilitates this process is lacking. There are three important questions that need to be further investigated and confirmed for a deeper understanding of this issue: (1) In human circulation, lyso-phospholipid can exchange its sn-1 FA to another non-esterified DHA through the following process: DHA is first incorporated into the sn-2 position of the glycerol backbone via acyltransferase activity, followed by phospholipase A1-mediated release of the original FA from the sn-1 position (49, 50). A critical question remains as to whether LDGTS undergoes similar enzyme-catalyzed processes analogous to the phospholipid metabolism. If confirmed, this mechanism could potentially replace the bound FA (mostly EPA) with DHA, thereby establishing LDGTS as an optimal exogenous DHA carrier *in vivo*. This hypothesis warrants further investigation; (2) Despite the potential ability of LDGTS to cross the BBB, direct experimental evidence is currently lacking. Therefore, further work should utilize more precise experimental models, such as Mfsd2a-expressing cell lines, to evaluate LDGTS plasma membrane permeability; and (3) On this basis, if LDGTS or LDGTS-containing microalgae oil becomes an important carrier for supplementing brain DHA, it is crucial to clarify the metabolic pathways of LDGTS/DGTS in the brain.

It is noteworthy that the enhancement of brain DHA uptake by microalgae oil appeared to be contingent on dosage. A higher intake of microalgae oil is not guaranteed to proportionally increase the brain's efficiency in absorbing DHA. The underlying mechanism responsible for this effect is unclear. It is plausible that there exists an optimal dosage threshold, surpassing which may not further augment the brain's DHA uptake capacity and could potentially diminish it due to excessively elevated fatty acid levels. Further research is needed to delineate the specific dynamics at play and to identify the precise dosage that maximizes DHA uptake by the brain without causing any adverse effects.

While our results provide promising insights, it is important to acknowledge that the modest sample size per treatment group (*n* = 4 or 5) may limit the statistical power to detect smaller effects. This could affect the robustness of certain conclusions, particularly for subtler phenotypic or biochemical differences. Future studies with larger cohorts would help validate these preliminary findings and enhance their translational relevance.

## Data Availability

The original contributions presented in the study are publicly available in the Science Data Bank. This data can be found here: https://doi.org/10.57760/sciencedb.26958
